# Comprehensive Analysis of Immune Cell Infiltration of m6a-Related lncRNA in Lung Squamous Cell Carcinoma and Construction of Relevant Prognostic Models

**DOI:** 10.1155/2022/9139823

**Published:** 2022-07-14

**Authors:** Pengfei Zhang, Huawei Li, Xiaoguang Cheng, Wei Wang

**Affiliations:** ^1^Department of Thoracic Surgery, The Second Affiliated Hospital of Harbin Medical University, Harbin 150000, China; ^2^Department of Thoracic Surgery, The First Hospital of Qiqihar, Qiqihar 161000, China; ^3^Department of Respiratory Medicine, The First Hospital of Qiqihar, Qiqihar 161000, China

## Abstract

Lung squamous cell carcinoma (LUSC) is the main cause of cancer-related mortality. Some studies demonstrate that m6a and long noncoding RNA (lncRNA) are vital in the pathogenesis of LUSC. In this study, we aimed to further understand the prognostic value of m6a-related lncRNAs in LUSC and their role in the immune microenvironment. For this, we obtained LUSC transcriptome and clinical data from the TCGA database. Further, the identified m6a-related and prognostically relevant lncRNAs were clustered into groups based on prognostic lncRNA expression. Further analysis of the differences between clusters was performed. Five m6A-related lncRNAs were used for model construction using the LASSO regression. The receiver-operating characteristic curve (ROC curves) and decision curve analysis (DCA) were used to assess the model accuracy. Finally, the model was validated using polymerase chain reaction (PCR). We identified 12 m6a-related lncRNAs that were associated with prognosis and were lowly expressed in tumors. Cytotoxic T-lymphocyte-associated protein 4 (CTLA4) highly correlated with prognostic genes, and differential analysis indicated that it was highly expressed in the tumor group and cluster 1. In cluster 2 TIME, tumor cells were less pure and more immune, and stromal-associated cells were present. A prognostic model was constructed based on five m6a-lncRNAs. The area under the curve (AUC) was >0.5 in test group and train group. The PCR results showed that the genes in the prognostic model were lowly expressed in the tumor and were statistically significant (*p* < 0.05). We noted that m6a-lncRNAs were strongly associated with LUSC prognosis and the immune microenvironment. Thus, PRC1-AS1, AL132780.2, AC013731.1, SNHG30, and AL358472.2 can be considered as new targets for the treatment of patients with LUSC.

## 1. Introduction

Lung cancer is the leading cause of cancer mortality worldwide, with a 5-year survival rate of 16–20%. Non-small-cell lung carcinoma accounts for four-fifths of the pathological lung cancer types, and lung squamous cell carcinoma (LUSC) is its subtype [1, 2]. LUSC often occurs in males that smoke and is characterized by a high recurrence rate, poor prognosis, and poor response to treatment [3, 4]. Since current therapeutic measures are not adequate, the search for prognostic biomarkers to prolong the survival of patients is crucial.

M6a, located at the sixth N-atom of adenine, is the most abundant methylation modification of RNAs (mRNAs) and ncRNAs, more than 60% of RNA modifications [2]. M6a is mainly regulated by m6a-related regulators such as methyltransferases, signal transducers, and demethylases [5]. In mammals, m6a modification, similar to DNA and protein modification, is a dynamic and reversible process [6]. The methylation level of m6a is associated with immune response, stem cell differentiation, embryonic development, and other processes that are vital in the cancer development [7].

lncRNAs are known to be greatly involved in the pathogenesis of LUSC. For instance, LINC00511 affects LUSC proliferation and metastasis by regulating miR-150-5p and TADA1 [8]. Downregulation of lncRNA PTTG3P is a predictor of poor prognosis in LUSC [9]. Moreover, the interaction between m6a modification and noncoding RNA is vital in the progression of cancer [10]. For instance, HBXIP upregulates METTL3 by inhibiting let-7g (that accelerates the proliferation of breast cancer cells) [11]. However, little is known about the mechanism of action of m6a-associated lncRNAs in LUSC.

We, therefore, investigated the prognostic influence of m6a-related lncRNAs, the correlation of CTLA4 and TIME in LUSC. Moreover, we aimed to understand the pathogenesis of LUSC as well as provide new treatment options.

## 2. Materials and Methods

### 2.1. Data Resource

**Figure 1:**
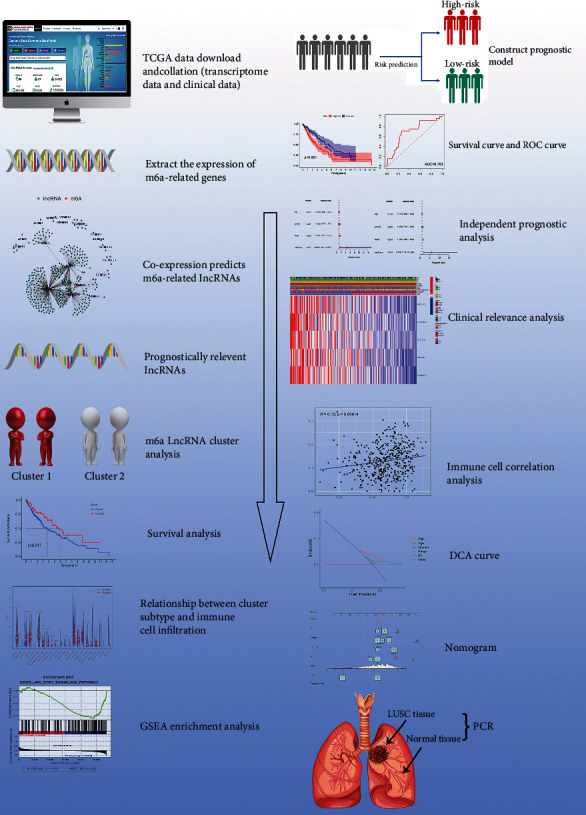
Main process.

The main flow of the article is shown in the Figure 1. We downloaded the LUSC RNA-seq transcriptome (FPKM) and clinical data from TCGA. These included 502 tumor tissue samples and 49 normal tissue samples. We employed mRNA matrices using the PERL software (Fig. https://www.perl.org/) and their corresponding scripts to organize transcriptome data and convert the IDs of genes. The same software and specific scripts were used for clinical data management as instructed by the developers.

#### 2.1.1. Identification of m6a-Related lncRNAs

We constructed a gene expression matrix and human configuration file that distinguishes mRNA and lncRNA using PERL software. By consulting the literature, we found 23 m6a-related regulators. Expression data for m6a-related genes were extracted using “limma” in R-software. Additionally, the m6-related lncRNAs was found by coexpression analysis, Corfilter > 0.4 and *p* < 0.01 as filtration conditions. The process uses the “limma” package in R-software (4.0.5). Next, the “igraph” package in R-software was used to draw the lncRNA coexpression network with m6a. The “limma” package in R-software was used to merge m6a-related lncRNA expression with survival data. Prognosis-related lncRNAs were found using the “survival” package in R-software (*p* < 0.05). The “limma,” “reshape2,” and “ggpubr” packages of R-software were used to distinguish the expression differences of prognosis-related lncRNA in normal tumors, and boxplot was plotted (*p* < 0.05).

#### 2.1.2. Role of m6a-Related lncRNAs

First, the “ConsensusClusterPlus” and “limma” packages were used to classify the samples into two subtypes according to their lncRNA expression (clusterAlg = ^‘^km′ and clusterNum = ^‘^2′). To analyze whether there is difference in survival between clusters and draw survival curve, we used the “survival” and “survminer” packages. For analyzing lncRNA expression differences as well as correlation with clinical features, a heat map was drawn using “pheatmappack”-age. Next, the CTLA4 expression of between tumors and normal tissues were analyzed among different clusters using the “limma,” “ggplot2,” and “ggpubr” packages in the R software. Furthermore, we explored the existence of a coexpression relationship between CTLA4 and prognostically relevant lncRNAs using the “survival” package in R software. When *p* < 0.05, the difference was considered statistically significant.

#### 2.1.3. Role of Immune Cell Infiltration and Tumor Immune Microenvironment (TIME)

With the algorithm of immune cell infiltration, we used “PreprocessCore,” “limma,” and “e1071” packages to convert the expression matrix of genes into a file of immune cell content. We performed the stromal, immune, and ESTIMATE scores on each sample to analyze the tumor microenvironment. Further, we performed immune cell differential analysis and plotting of violin map expression using the “limma” and “vioplot” packages in R software. Finally, we divided the differential immune cells in the two clusters. The immune, ESTIMATE, and stromal scores analyzed in the two clusters were using the “limma” package in R software. Gene set enrichment analysis (GSEA) (https://www.gseamsigdb.org/gsea/index.jsp) were collated using PERL software. Active functions and pathways based on GSEA software screening criteria for FDR *q* value and FWER (*p* < 0.05).

#### 2.1.4. Development of the m6a-lncRNA-Related Prognostic Model

Prognostic models were constructed using the LASSO regression. The risk score was calculated using the formula: Risk scores = *Pn* *Xi* × *Yi* (*X*: coefficient of each gene, *Y*: expression of each gene). The samples were divided into the test and train groups in a 7 : 3 ratio. The comparison of all samples with the median value of the risk score of the prognostic m6a-lncRNAs was divided into low- and high-risk groups. The survival curves for high-risk and low-risk groups were compared. To assess the accuracy of the prognostic model, the 1-year ROC curve was obtained using the “timeROC” package. Risk curves were plotted to evaluate the survival status and risk associated with m6a-lncRNA. Through independent prognostic analysis, we analyzed the independent prognostic factor of our model. Univariate and multivariate analyses were performed, while hazard ratios were calculated. We equally validated the utility of the model in different clinical groups. Moreover, we assessed the expression of CTLA4 in different risk groups using the “survival” and “survminer” packages in R-software through differential analysis of genes. The relationship between immune cells and risk scores was illustrated using scattered plots through immune cell correlation analysis. Moreover, the decision curve analysis (DCA) and nomogram were drawn using the “ggDCA” package.

#### 2.1.5. Samples and Quantitative Real-Time Polymerase Chain Reaction (qRT-PCR)

We collected 25 pairs of LUSC and normal lung tissues from patients who underwent surgical treatment at the Department of Thoracic Surgery of the Second Affiliated Hospital of Harbin Medical University between February and March 2011. Fresh LUSC and normal lung tissues were frozen and stored at -80°C. The study was approved by the Medical Ethics Committee of the Second Affiliated Hospital of Harbin Medical University, and samples were collected in accordance with the approved guidelines. Informed consent was acquired from each participant. The specific primer sequences (Table 1) used in this experiment were designed and synthesized by the Shanghai Xinbei Biological Company.

## 3. Results

### 3.1. Identification of m6a-Related lncRNAs in LUSC Patients

**Figure 2:**
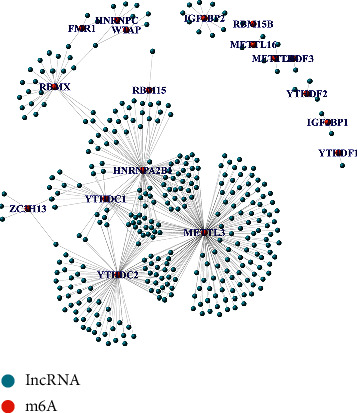
Network diagram of coexpression of m6a gene and lncRNA.

First, LUSC transcriptome data were downloaded through the TCGA database. The transcriptome data were divided into mRNA and lncRNA. We obtained 1,407 lncRNAs and 23 m6a-related regulators via literature search. The expression of m6a-related regulators was extracted from the transcriptome data. We obtained m6a-related lncRNA through coexpression methods. We obtained 336 lncRNAs significantly correlated with m6a-related regulators. The m6a-related lncRNA coexpression network in lung squamous cell carcinoma is illustrated in Figure 2.

### 3.2. Significant Correlation of Consensus Clustering for m6a-Related lncRNA Methylation Regulators with the Characteristics and Survival of Patients with LUSC

**Figure 3:**
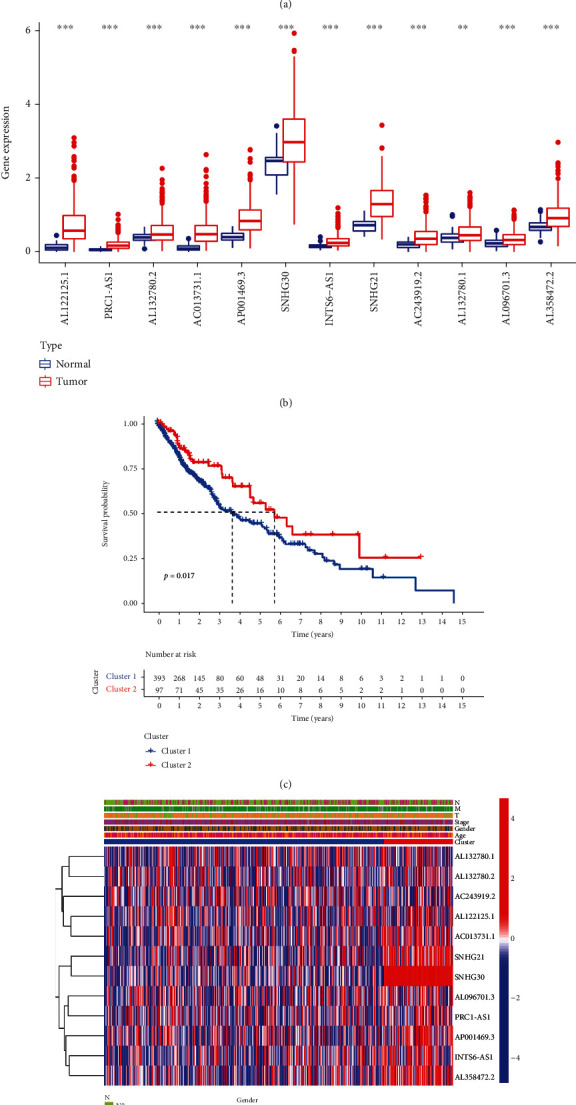
Expression of m6a-related lncRNA and its prognostic role in lung squamous cell carcinoma. (a) Prognostic relevant lncRNAs were obtained by univariate Cox regression analysis. Forest plots were drawn, and confidence intervals and hazard ratios were calculated. Green indicates low risk. (b) Boxplot of differential expression of m6a-prognostic lncRNAs in the normal and tumors. *p* < 0.05; ^∗∗^*p* < 0.01; ^∗∗∗^*p* < 0.001. (c) They were grouped according to m6a-prognostic lncRNA expression clustering. Cluster 2 survived better than cluster 1, *p* = 0.017. (d) Heat map of the relationship between prognostic lncRNAs in different cluster expression and clinical characteristics. There was no correlation between prognostic lncRNAs and clinical characteristics.

First, we downloaded clinical data related to LUSC from the TCGA database, combining m6a-related lncRNA expression with survival data (we deleted samples with blank survival time or survival status in clinical data). The results of univariate Cox analysis were shown in forest plots, and all 12 m6A-related prognostic lncRNAs were noted to be low-risk genes (Figure 3(a)). The expression of all genes was significantly different between the normal and tumor groups (Figure 3(b)). Samples were classified by means of clustering based on the expression of prognostically m6a-lncRNA. We divided 490 patients with LUSC into two subtypes; cluster 1 (*n* = 393) and cluster 2 (*n* = 97). On comparing their survival rates, cluster 2 had a significantly better survival curve than that of cluster 1 (Figure 3(c)). We equally compared the clinical features using heat map. Clinical characteristics were similar in both the clusters (Figure 3(d)). However, prognostic m6a-lncRNAs were significantly overexpressed in cluster 2 than in cluster 1. This is consistent with the poor prognosis observed in cluster 1.

### 3.3. Association of CTLA4 with m6a-Related lncRNA

**Figure 4:**
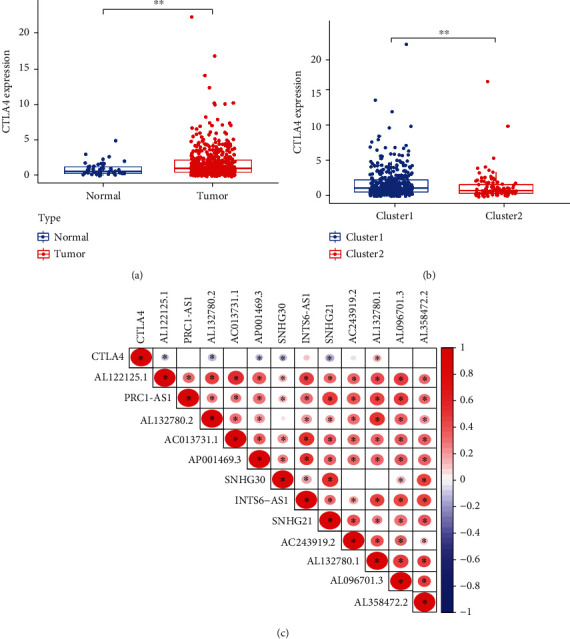
CTLA4 correlation analysis. (a) CTLA4 expression is different between tumors and normal (*p* < 0.01). (b) CTLA4 expression was higher in cluster 1 than cluster 2 (*p* < 0.01). (c) Analysis of the correlation between CTLA4 and prognostic m6a-lncRNA. Red represents a negative correlation, and blue represents a positive correlation. ^∗^Statistically significant.

We deduced the correlation between CTLA4 and m6a-related lncRNA by assessing the differences in the expression of the two groups. There was an increased expression of CTLA4 in the tumor group compared with normal tissue (Figure 4(a)). Moreover, CTLA4 was overexpressed in cluster 1 (Figure 4(b)). Gene correlation analysis revealed that CTLA4 was closely associated with m6A-related prognostic lncRNAs (Figure 4(c)).

### 3.4. Role of Immune Cell Infiltration and Tumor Immune Microenvironment (TIME)

**Figure 5:**
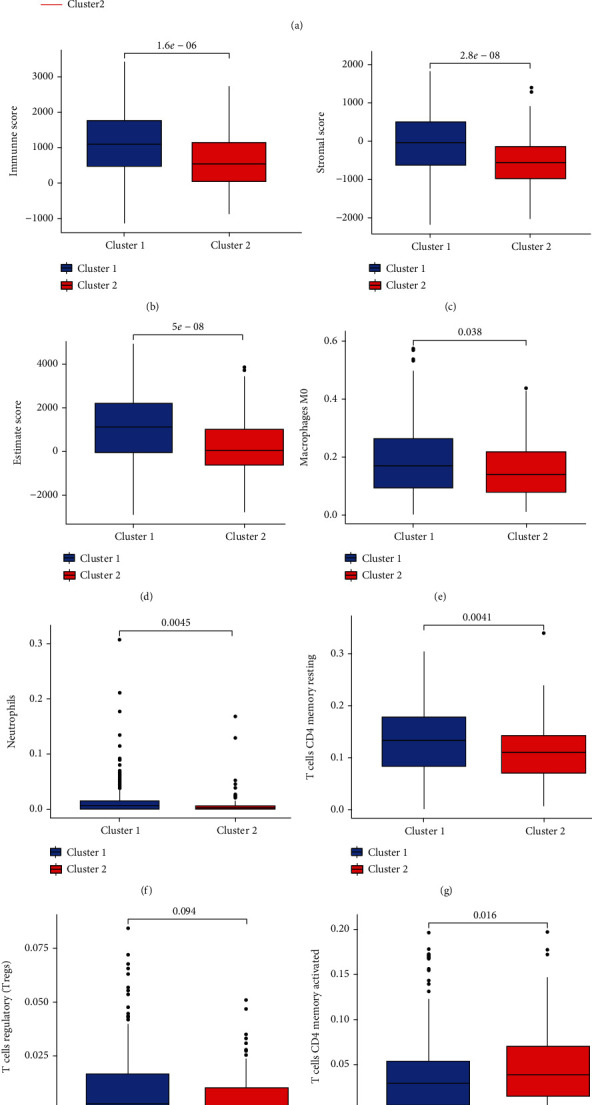
Correlation analysis of tumor immune microenvironment. (a) Differential analysis of immune cells in different clusters. (b–h) Boxplot indicated that immune cell infiltration was higher in cluster 1 than in cluster 2, *p* < 0.05. (i, j) Boxplot indicated that immune cell infiltration was higher in cluster 2 than in cluster 1, *p* < 0.05.

To further investigate the role of m6a-related lncRNA regulators in the TIME of LUSC, the immune cell infiltration between the two clusters was shown (Figure 5(a)). Based on the immune, stromal, and ESTIMATE scores, cluster 2 was greater than cluster 1 (Figures 5(b)–5(d)). This means that in cluster 2 TIME, the purity of tumor cells was lower, and there were more immune and stromal-related cells present. Next, the expression of the two clusters in 22 immune cells was analyzed. Cluster 1 showed higher infiltration levels of macrophages M0, neutrophils, CD4 T cells memory, and T regulatory cells (Tregs) (Figures 5(e)–5(h)). Cluster 2 showed higher infiltration levels of activated CD4 and CD8 T cells (Figures 5(i) and 5(j)). GSEA enrichment analysis was used to clarify the functions and pathways between the different clusters (Figure S1). An FDR *q* value and FWER *p* < 0.05 were used as criteria for filtration. The result showed that the most enriched pathway was the JAK-STAT-signaling-pathway. The activation of this pathway promotes tumorigenesis and cancer progression. Our results revealed that cluster 1 had a poor prognosis. Other pathways with positive correlations have been illustrated in S1.

### 3.5. m6a-Related Prognostic lncRNA Model

**Figure 6:**
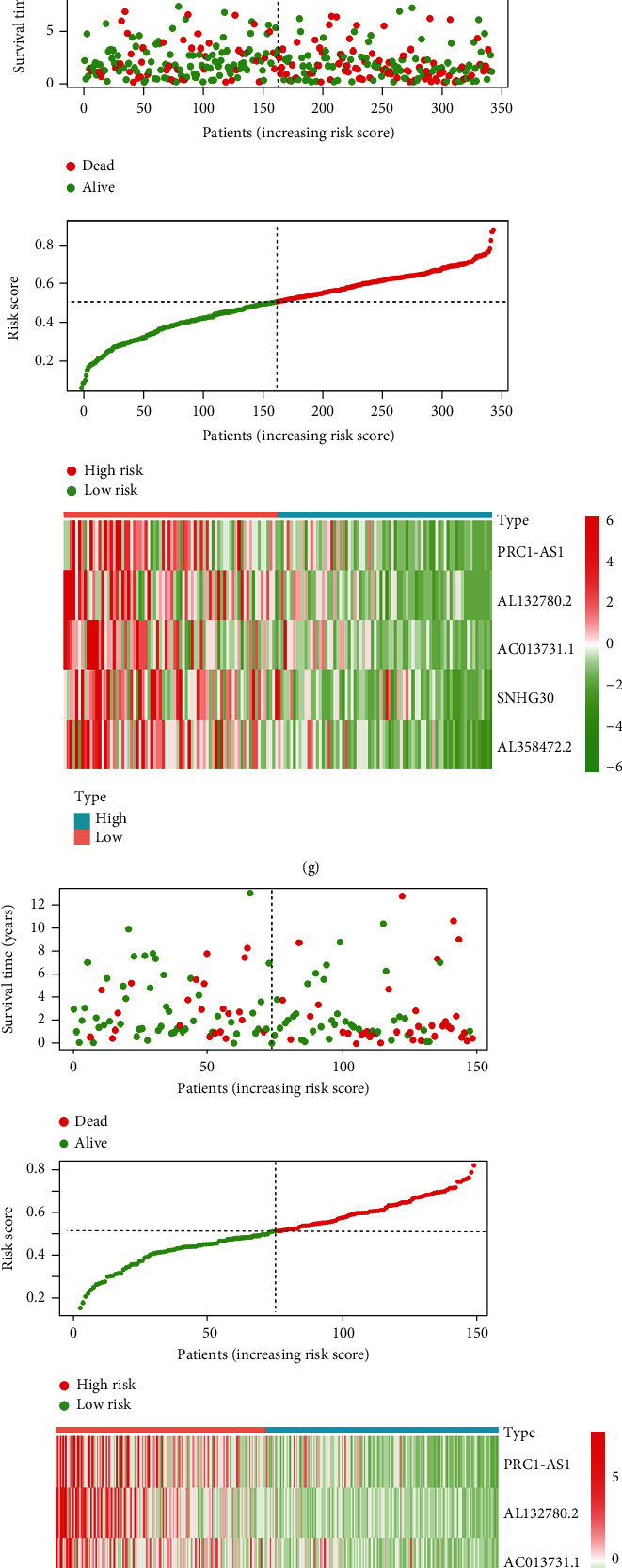
Construction of a prognostic model for patients with LUSC. (a, b) A prognostic model for LUSC patients was constructed by lasso regression. (c, d) High-risk group had lower survival rate than low-risk group ((c) test group; (d) train group *p* < 0.05). (e, f) Receiver-operating characteristics curve was used to predict the accuracy of the model ((e) test group area under the curve = 0.601; (f) train group area under the curve = 0.703). (g, h) Risk-related curve and risk-related spot plot indicate an increase in patient deaths with increasing risk score. Risk-related heat map indicates gene lower expression in high-risk group. This implies that these genes may be beneficial for the prognosis of LUSC patients ((g) test group; (h) train group). (i–l) Risk score can be used as an independent prognostic factor, in multivariate and univariate analyses. (i, j) Test group. (k, l) Train group. (i–k) Univariate analysis. (j–l) Multivariate analysis.

In this study, we explored the prognostic role of m6a-related lncRNA in patients with LUSC. Moreover, we randomly divided 490 patients into two groups; the test group with 342 patients and train group with 148 patients (at a ratio of 7 : 3). Each group was further divided into high-risk and low-risk groups according to the median value of the risk score of prognostic m6a-lncRNAs via the LASSO regression (Figures 6(a) and 6(b)). Survival plots showed lower survival in high-risk patients in both groups (Figures 6(c) and 6(d)). The accuracy of the model in predicting patient survival was evaluated using ROC curves (Figures 6(e) and 6(f)). However, the area under the curve (AUC) was >0.5 in both groups, indicating that the model had a high accuracy. Patients with high scores experienced shorter overall survival time. In high-risk patients, heat map showed lower expression of the five genes (Figures 6(g) and 6(h)). We equally performed univariate and multivariate Cox analysis for all data collected (Figures 6(i)–6(l)). It was found that the model was significant in both groups (*p* < 0.05) and can be used as an independent prognostic factor.

### 3.6. Risk Correlation Analysis

**Figure 7:**
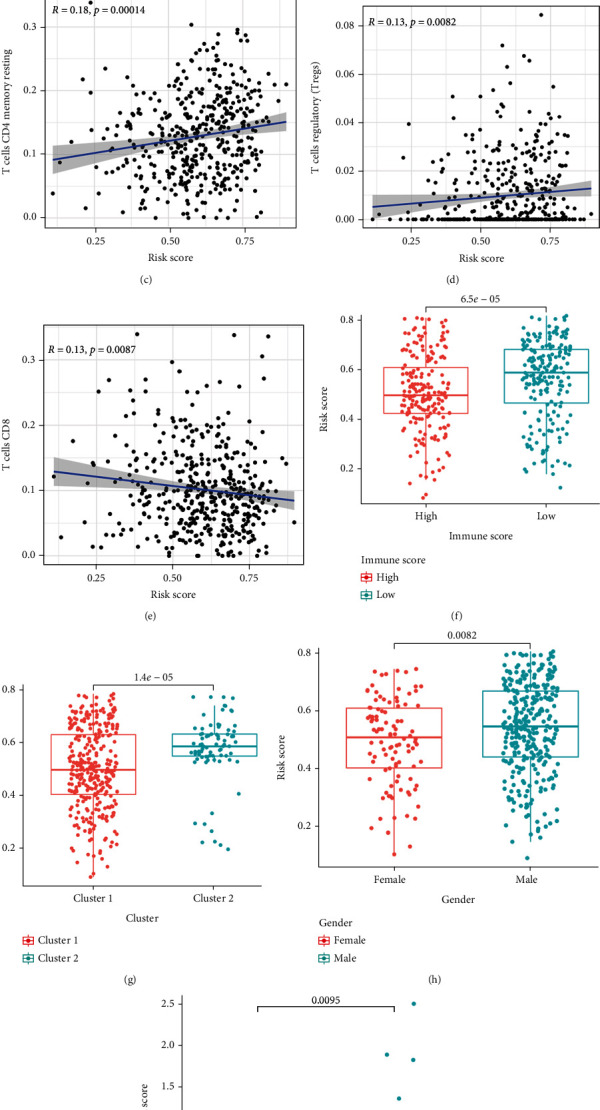
Risk correlation analysis. (a) CTLA4 expression increased in the high-risk group compared with the low-risk group, *p* = 0.0039. (b–e) Scatterplot indicates the relationship between risk score and immune cells. (f–i) Boxplot indicates correlation of risk scores with clinical characteristics, *p* < 0.05.

CTLA4 was overexpressed in the high-risk group relative to the low-risk group (Figure 7(a)). We found a positive correlation between risk and immune cells such as neutrophils, resting CD4 memory T cells, and Tregs (Figures 7(b)–7(d)). However, CD8 T cells had a negative correlation with risk (Figure 7(e)). This can be used to distinguish which immune cells are beneficial or harmful in lung squamous cell carcinoma. A low immune score was associated with a higher immune score (Figure 7(f)). Cluster 2 had a lower risk score, which is consistent with our previous study (Figure 7(g)). Male patients had a higher risk of poor prognosis than that in females (Figure 7(h)). Patient grade III had a higher risk than in grades I-II (Figure 7(i)).

### 3.7. Validation of Prognostic Models

**Figure 8:**
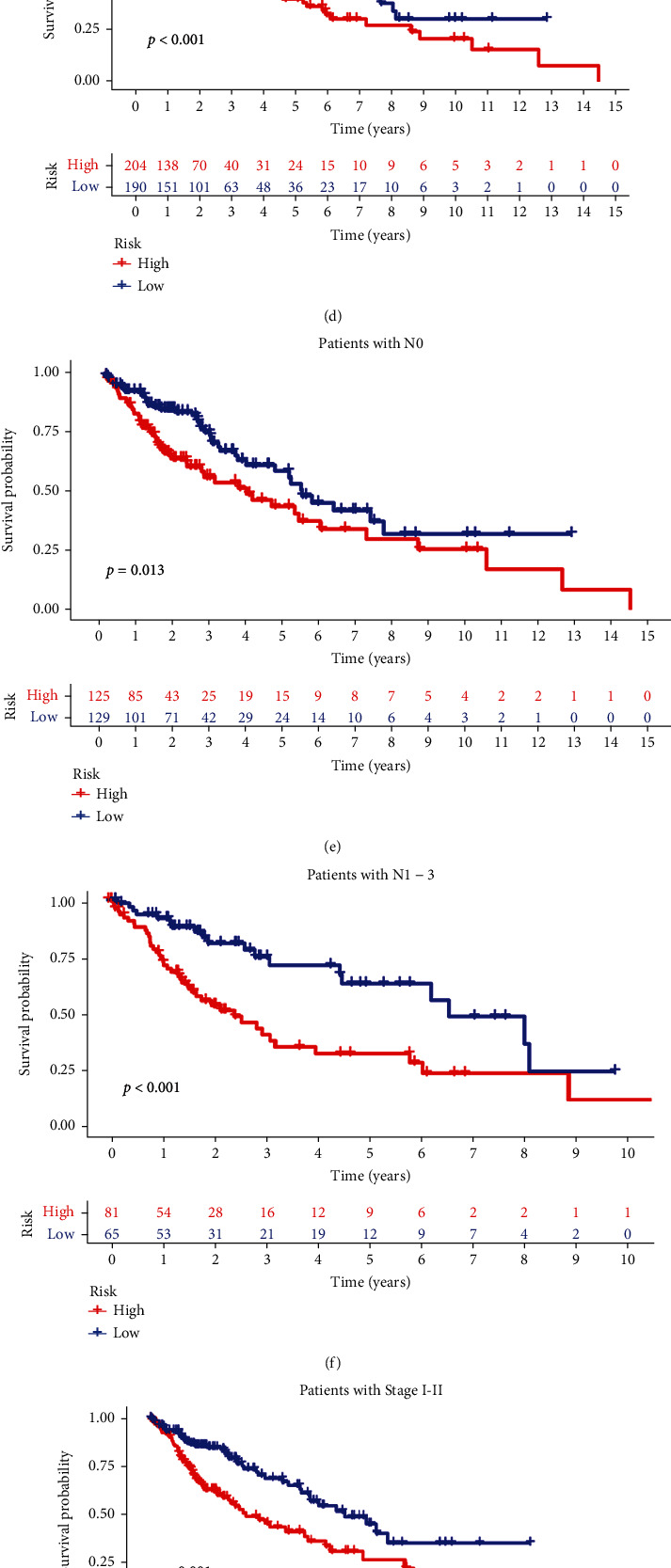
Validation of the model and prediction of patient survival. (a–h) This model is suitable for different clinical groups, *p* < 0.05. (i) The decision curve analysis shows that the risk is furthest from all the curves; predicting patient survival with models is superior to clinical traits. (j) Nomogram for predicting LUSC patient 1-, 3-, and 5-year overall survival.

**Figure 9:**
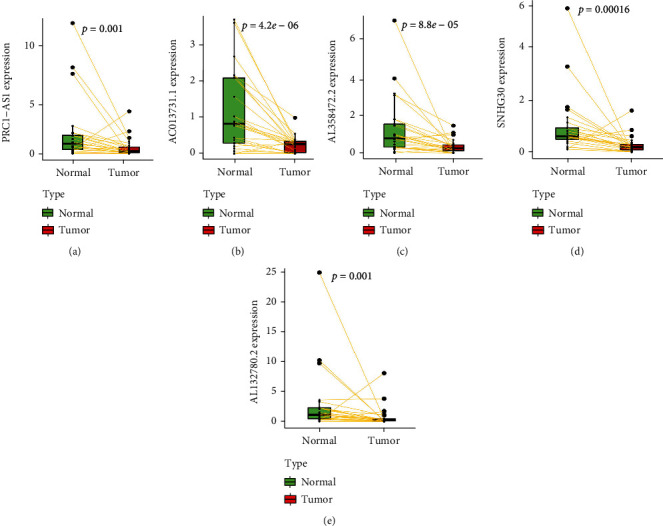
The model is validated by PCR. (a–e) Differential expression of lncRNA in normal cells and tumors.

It was found that the constructed prognostic model was significant in these clinical groups (Figures 8(a)–8(h)). Decision curves illustrated that predicting patient survival with this model is superior to other clinical traits (Figure 8(i)). This indicated that the prognostic model constructed by five m6a-lncRNA accurately predicts the prognosis of patients. A nomogram combining clinicopathological features and the risk score of patients can be used to predict the survival of patients (Figure 8(j)). To validate the expression levels of m6a-related prognostic lncRNAs in LUSC samples, an RT-qPCR was performed to examine the expression levels of the five lncRNA prognostic models. We found that these five lnc-RNAs (PRC1-AS1, AL132780.2, AC013731.1, SNHG30, and AL358472.2) were underexpressed in the tumoral group. These genes were significantly different in the tumor and nontumor group, *p* < 0.05 (Figure 9). This was consistent with the previous findings.

## 4. Discussion

**Figure 10:**
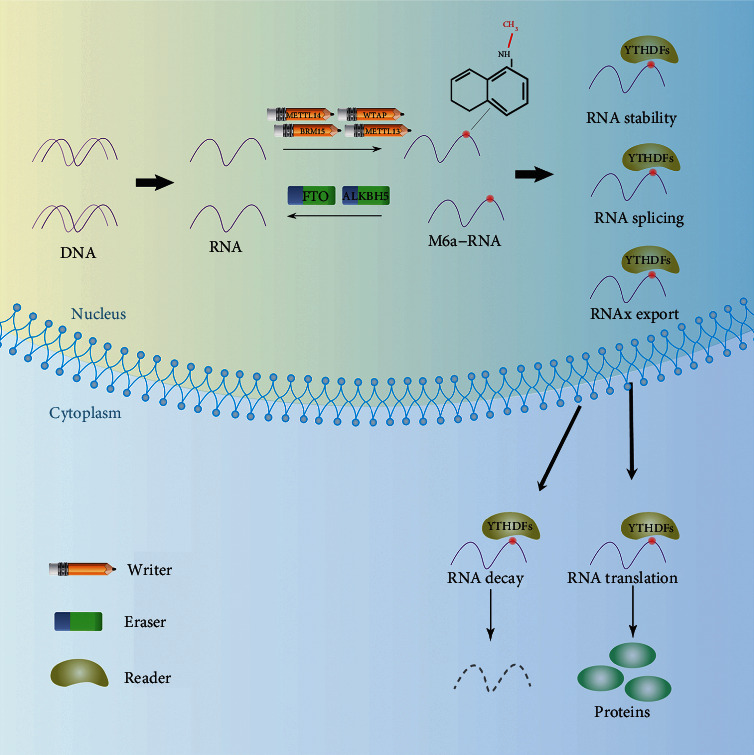
Dynamically reversible process of m6a RNA methylation mediated by regulators (“erasers,” “writers,” and “readers”).

Lung cancer is the leading cause of cancer mortality worldwide with an ever-increasing incidence [12]. Since symptoms are not obvious in the early stage, the rate of early detection is very low. Moreover, treatment methods are limited, resulting in very low five-year survival rates [13]. However, recent treatment options, targeted therapy and immunotherapy, have promising effects against lung cancer [14]. Recently, it has been found that m6a is closely related to cancer and promotes the renewal of cancer stem cells [15]. Moreover, m6a is a sensitive marker for the diagnosis, treatment, and prognosis of lung cancer [16]. N6-methyladenosine (m6a) is the main mode of internal modification of lncRNAs and is vital in their processing, transport, and stability [17]. There are three main m6a-related regulators; methyltransferase (writers: METTL3, METTL14, METTL16, WTAP, VIRMA, ZC3H13, RBM15, and RBM15B), binding protein (readers: YTHDC1, YTHDC2, YTHDF1, YTHDF2, YTHDF3, HNRNPC, FMR1, LRPPRC, HNRNPA2B1, IGF2BP1, IGF2BP2, IGF2BP3, and RBMX.3), and demethylase (erasers: FTO and ALKBH5) [6, 18]. The dynamically reversible biological processes in RNA via the regulator m6a are summarized in Figure 10. In recent years, studies have found that lncRNAs are vital in the diagnosis and development of lung cancer [17]. Previous studies have focused on monogenic m6a-related genes. However, the development and progression of cancer is regulated by multiple genes; so, it is more meaningful to systematically study the role of m6a-related lncRNAs in LUSC.

In this study, LUSC transcriptome data was first downloaded from the TCGA dataset, and lncRNA data was extracted from the transcriptome data. The expression levels of m6a-related genes were extracted; m6a-related lncRNAs were found by coexpression analysis.

Moreover, 12 m6a-related lncRNAs were associated with prognosis through univariate Cox regression analysis. The expression of these 12 m6a-related lncRNAs was different in the two groups, and all were underexpressed in the tumor group (*p* < 0.05). Samples were clustered based on the expression of lncRNAs using an algorithm for clustering. Sample survival was found to vary between clusters (*p* < 0.05). This shows that these lncRNAs are involved in the development and progression of LUSC. Previous studies have demonstrated that m6a-related genes are vital in the pathogenesis of lung cancer. The m6a demethylase FTO affects the growth of lung cancer by regulating USP7 m6a methyltransferase [19]. Mettl3 is equally vital in the pathogenesis of LUSC as it regulates miR-143-3p/VASH1 axis [20]. However, little is known about m6a-related lncRNAs.

CTLA4 is the first immune-checkpoint receptor in clinical practice. CTLA4 is expressed exclusively on T cells and regulates T-cell activation [21–23]. Immunotherapy for cancer is mainly based on the immune escape theory of cancer cells [24]. CTLA4 antibodies are the first immunotherapeutic drugs to be designed [24]. In this study, CTLA4 was found to be overexpressed in the tumor group than in the normal group. Expression was increased in cluster 1. This was consistent with previous results. In the relationship between CTLA4 and risk score, CTLA4 expression was higher in the high-risk group. We noted that CTLA4 was correlated with prognostically relevant m6a-lncRNAs and negatively correlated with AL122125.1, AL132780.2, AP001469.3, SNHG30, and SNHG21. Moreover, these findings revealed that genes have a cancer-inhibiting effect. This is consistent with our previous hypothesis and provides novel loci for immunotherapy and targeted therapies.

Further investigations revealed that macrophages M0, neutrophils, CD4 memory T cells, and T cells regulatory (Tregs) were increased in cluster 1, while activated CD4 and CD8 T cells increased in cluster 2. Neutrophils, T cells CD4 resting memory, T cells CD8, and T cells regulatory (Tregs) were associated with the prognosis of LUSC in TIME, and immune cells have shown their utility in predicting the prognosis of patients with LUSC. An increase in the number of neutrophils often results in a poor prognosis for patients [25]. Regulatory T cells play an immunosuppressive role by expressing the transcription factor FoxP3 and promoting tumor progression by inhibiting the antitumor immune response [26]. These findings suggest that m6a-lncRNA regulates TIME in LUSC to some extent. Further investigations on the ESTIMATE and Stroma scores in two clusters of LUSC showed that they were higher in cluster 1. Tumor purity was lower, and immune-related cells infiltrated more in cluster 1. High immune scores tended to have low risk in other tumors. However, high immune scores were associated with a high risk in LUSC. Further studies are needed to ascertain this point.

A prognostic model based on five prognostically relevant m6a-lncRNAs was established. We randomly divided data collected into training and test groups with a 3 : 7 ratio, respectively. The high-risk group exhibited a lower survival rate. This shows that the model can predict the prognosis of patients. Moreover, it can also affect the prognosis independently from others. Furthermore, decision curve analysis (DCA) results indicate that predicting the prognosis of patients with this model is superior to other clinical traits. The nomogram predicts the survival of patients based on their clinical traits and risk scores. Finally, the model was validated using PCR. We noted that the five lncRNAs in the constructed model were underexpressed in the tumor. This was consistent with previous findings that m6a modification may affect the structure of lncRNAs and, thus, affect binding to proteins [27, 28]. The interaction between m6a methylation and lncRNAs affects the proliferation, invasion, and metastasis of cancer cells [29]. Thus, a review of current literature suggests that m6a-lncRNA can be used as an independent prognostic model for patients with LUSC.

## 5. Conclusion

The LUSC transcriptome and corresponding clinical data were obtained through the TCGA database. We found that 336 lncRNAs were closely related to m6a by coexpression analysis, and we obtained 12 m6a-related lncRNAs associated with prognosis using univariate Cox analysis (*p* < 0.05). Consensus cluster of these lncRNAs showed that different LUSC subtypes (cluster1/2) exhibited different survival times (*p* < 0.05) and TIME (*p* < 0.05). It was found that CTLA4 was highly correlated with m6a-related lncRNAs, and differential analysis indicated that CTLA4 was associated with the prognosis of patients with LUSC (*p* < 0.05). The prognostic model constructed by 5 m6a-related lncRNAs (PRC1-AS1, AL132780.2, AC013731.1, SNHG30, and AL358472.2) can accurately predict the prognosis of patients (AUC > 0.5). These results suggest that m6a-related lncRNAs may be associated with the development of LUSC and its TIME. This may provide new treatment options for immunotherapy, targeted therapy, and the provision of new prognostic biomarkers.

## Figures and Tables

**Table 1:** Primer sequences.

List of oligonucleotide sequences	5′→3′
*Primers for qRT-PCR*	
PRC1-AS1-F	CTTCCTGAATAAGACAACGTACCAC
PRC1-AS1-R	AAAAGGTAGGCATAGATGGTGC
AL132780.2-F	CCGCTGTGAAGTCCAGTTCT
AL132780.2-F	CTTCTCCCTTTGGTTTTGGTCC
AC013731.1-F	AATAGTGGTCTTATTGTATGGCTGG
AC013731.1-R	TTCTCTGGTTTGAATGCCTCTG
SNHG30-F	GTTCGTGGGATTTGGACCTTAG
SNHG30-R	CCATACCTCAAGCACCTCCAAG
AL358472.2-F	TGGTTTCTCTGACATCCTTCCC
AL358472.2-R	TCATGTTCGTCATGTGTCTCGG
GAPDH-F	GAGAAGTATGACAACAGCCTCAA
GAPDH-R	GCCATCACGCCACAGTTT

## Data Availability

Publicly available datasets were analyzed in this study, the LUSC RNA-seq transcriptome (FPKM) and clinical data from TCGA website (https://portal.gdc.cancer.gov/).

## Supplementary Materials

Supplementary Materials Supplementary Figure 1: GSEA enrichment analysis.
