# Supplementary figures and images for: Comprehensive Analysis of Immune Cell Infiltration of m6a-Related lncRNA in Lung Squamous Cell Carcinoma and Construction of Relevant Prognostic Models
Source: Biomed Res Int. 2022 Jul 14;2022:9139823. doi: 10.1155/2022/9139823 (PMC9303127; doi:10.1155/2022/9139823)

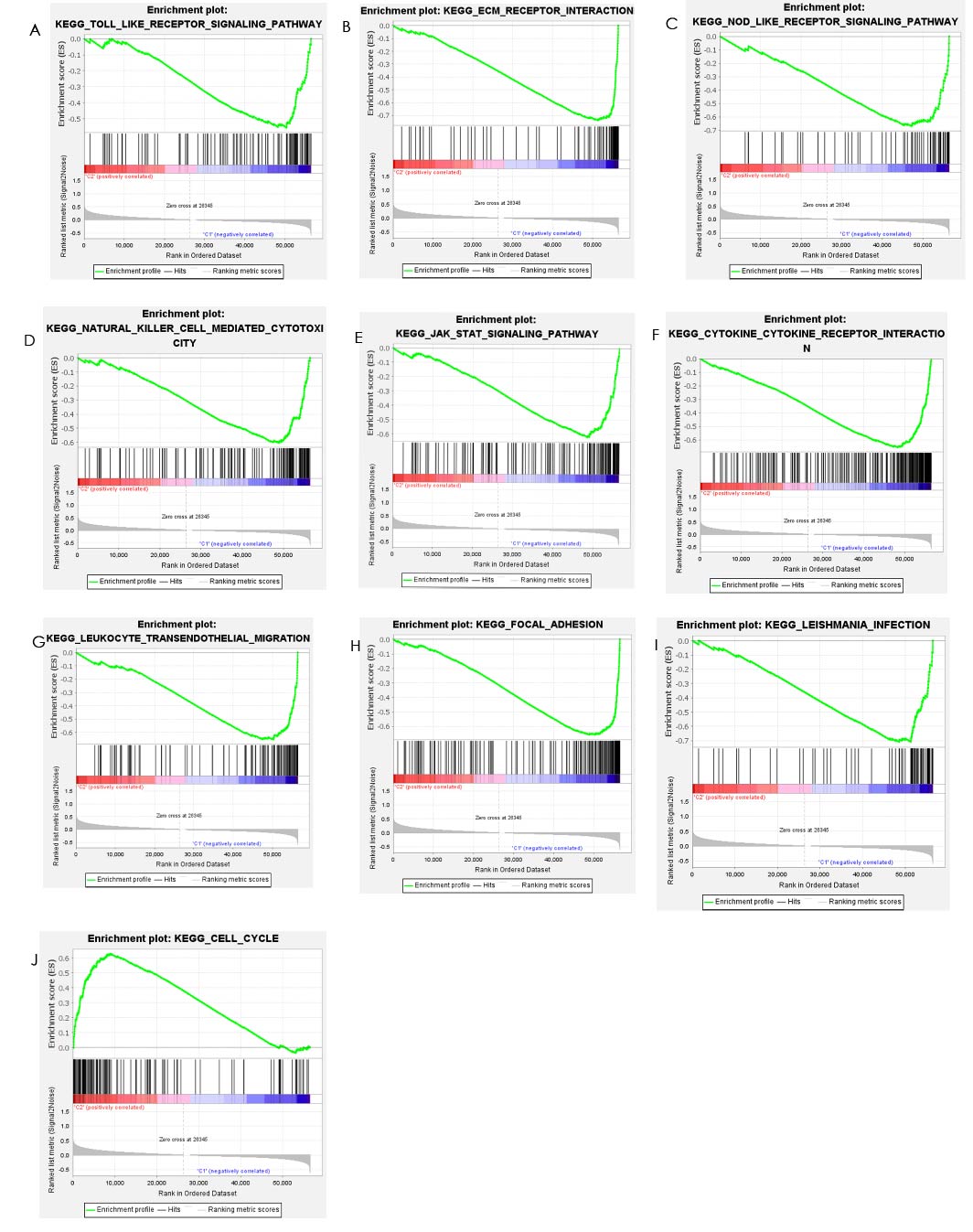

Supplement: Supplementary Materials — Supplementary Figure 1: GSEA enrichment analysis. [file 9139823.f1.jpg]
